# Sensory Changes Related to Swallowing in Motor Neurone Disease

**DOI:** 10.1007/s00455-024-10742-x

**Published:** 2024-08-03

**Authors:** Megan Paterson, Sebastian Doeltgen, Rebecca Francis

**Affiliations:** 1 Speech Pathology, College of Nursing and Health Sciences, Flinders University, Bedford Park South Australia 5042, GPO Box 2100, Adelaide, SA 5001 Australia; 2 Swallowing Neurorehabilitation Research Laboratory, Caring Futures Institute, Flinders University, Adelaide, Australia

**Keywords:** Motor neurone disease, Amyotrophic lateral sclerosis, Dysphagia, Deglutition, Sensation, Sensory function

## Abstract

Dysphagia is common in motor neurone disease (MND) and associated with negative health and psychosocial outcomes. Although largely considered a motor disease, a growing body of evidence suggests that MND can also affect the sensory system. As intact sensation is vital for safe swallowing, and sensory changes can influence the clinical management of dysphagia in people living with MND, this review evaluated and summarised the current evidence for sensory changes related to swallowing in MND. Of 3,481 articles originally identified, 29 met the inclusion criteria. Of these, 20 studies reported sensory changes, which included laryngeal sensation, taste, gag reflex, cough reflex, tongue sensation, smell, palatal and pharyngeal sensation, silent aspiration, and undefined sensation of the swallowing mechanism. Sensory changes were either described as decreased (*n* = 16) or heightened (*n* = 4). In the remaining nine studies, sensory function was reported as unaffected. The presence of changes to sensory function related to swallowing in MND remains inconclusive, although an increasing number of studies report sensory changes in some sensory domains. Future research is needed to evaluate the prevalence of sensory changes in MND and how such changes may influence dysphagia and its management.

## Introduction

Motor neurone disease (MND) is an umbrella term for a series of neurodegenerative, life limiting diseases [1]. MND is hallmarked by progressive loss of motor function as the result of degeneration of both upper and lower motor neurones [1–3]. MND is largely considered a motor disease, however, emerging evidence suggests non-motor deficits, including changes in sensory function, may also be present [4–8].

It is estimated that over 85% of people living with MND (plwMND) experience dysphagia [9, 10], with others suggesting almost all plwMND will develop dysphagia as the disease progresses [11, 12]. Aspiration is a frequently reported consequence of dysphagia in plwMND [2, 8, 10, 13], and can contribute to the leading causes of death within this population, i.e. pneumonia or hypoxia [14, 15]. The effects of dysphagia extend beyond aspiration, however. Dysphagia is associated with poorer hydration and nutrition [16, 17] and is also linked to negative psychosocial outcomes, with plwMND experiencing a strong fear of choking and diminished eating related pleasure [16]. In addition, reduced quality of life [18] and increased health care expenditure [19] have also been reported. As such, early identification and management of functions related to swallowing is critical.

Swallowing is a complex sensorimotor process that requires precise sensorimotor neural control to accomplish safe and efficient bolus passage through the pharynx [20, 21]. As sensory function heavily integrates with and modulates motor function [2, 22], it is critical to understand the functioning of both sensory and motor systems when diagnosing and managing dysphagia [2]. Of note, however, sensory function may not be considered in the assessment and management of dysphagia in plwMND. The current diagnostic model for MND is largely based on clinical presentation, in which sensory changes may be viewed as exclusionary for a diagnosis [1, 13, 23]. Anecdotally however, plwMND have reported sensory symptoms for some time, including neuropathic pain, tingling, and diminished temperature sensation [4, 24–26]. In the distal limbs, sensory changes have been observed and recorded in multiple studies [27–29]. Sensation involved in swallowing, such as olfactory dysfunction, has also been reported; however, its links to dysphagia have not been evaluated in plwMND [30, 31]. These findings indicate that MND may affect sensory function relevant for safe and efficient swallowing. The aim of this scoping review was to comprehensively map and summarise the existing literature in this context.

## Methods

We conducted a scoping review following an established framework [32] to explore what is known about changes in sensation related to swallowing in MND. This approach enabled the inclusion of diverse study designs to allow for a holistic synthesis and appraisal of the existing literature.

### Search Methods and Inclusion Criteria

Two preliminary searches were conducted in April and May 2022, followed by two searches in May 2022 and April 2023 with revised search terms across seven databases (Scopus, Medline, Emcare, CINAHL, Web of Science, Proquest, PsycInfo). Key words related to MND, swallowing and sensation are shown in Table 1. Terms relating to children, animals, and genetics were excluded from the search. In addition, hand-searching of the reference lists of included studies was undertaken to identify additional relevant studies. Key studies known to the research team were also included. All reviewers followed a shared inclusion and exclusion criteria decision tree. To be included, a study had to (i) report on MND or its subtypes, (ii) mention dysphagia or related terms, and (iii) mention sensory changes related to swallowing. Studies were not required to explicitly investigate sensory changes in MND; a statement on the presence or absence of sensory change in the context of plwMND was sufficient for inclusion. Studies were excluded if a full text was not available in English, or if it did not discuss any of the relevant inclusion criteria.

**Table 1:** List of search terms

Search terms related to MND, swallowing, and sensation				
Motor neurone disease Motor neuron disease Amyotrophic lateral sclerosis Progressive Muscular Atrophy Progressive Bulbar Palsy Primary Lateral Sclerosis Kennedy’s disease	**AND**	Sensation Senso* Tactile Tast* Gustation Smell* Olfaction Afferent Special sensory Special sensation General sensory General sensation Cough	**AND**	Dysphag* Swallowing disorder Swallowing impair* Eat* Swallow* Drink* Deglut* Deglut* disorders Meal* Feed* Laryn* Pharyn* Oral Mouth Tongue Lips

Search terms used across Scopus, Medline, Emcare, CINAHL, Web of Science, Proquest, and PsycInfo

Title and abstract screening was undertaken on the online platform Covidence [33] by two reviewers who were blind to each other’s ratings. Conflicts were resolved by consensus following discussion. Subsequently, the full texts of the remaining studies were assessed for eligibility using the same screening approach.

### Data Extraction

Data from included studies were extracted and summarised in a Microsoft Excel spreadsheet, which was discussed and iteratively refined by the research team. The following information was extracted:

1. Study: authors, year of publication, title, journal, study location, study type, study aims, and methodology.
2. Participants: sample size, age and gender demographics, status of MND and dysphagia diagnosis, exclusion criteria, and sample baselines.
3. Outcome measures: sensory function measured, assessment tools used, presence of sensory change, description of sensory change, primary and/or secondary statements regarding sensory change, a summary of the results, and author recommendations.

### Data Analysis

We conducted a descriptive thematic analysis guided by the Braun and Clark framework [34]. Extracted data were grouped into broad characteristics to assist in accurately illustrating the evidence gained from included studies. The data analysis process was iteratively refined, in that characteristics were cyclically reviewed and modified to be most representative of the data. We arrived at the final characteristics by evaluating which data would be necessary to establish a comprehensive narrative of what is currently known about sensory changes related to swallowing in MND. Furthermore, we also provide additional characteristics that assist in portraying not only what is being reported, but also the context in which this information is being conveyed.

### Evidence Grading

We conducted evidence quality analysis of the included studies using the Grading of Recommendations, Assessment, Development and Evaluation (GRADE) framework [35]. This framework assesses evidence quality across four levels: very low, low, moderate, and high. The quality rating of each primary study was determined by taking the individual methodological rigour into consideration. Ratings were discussed and agreed upon by the research team.

## Results

**Fig. 1:**
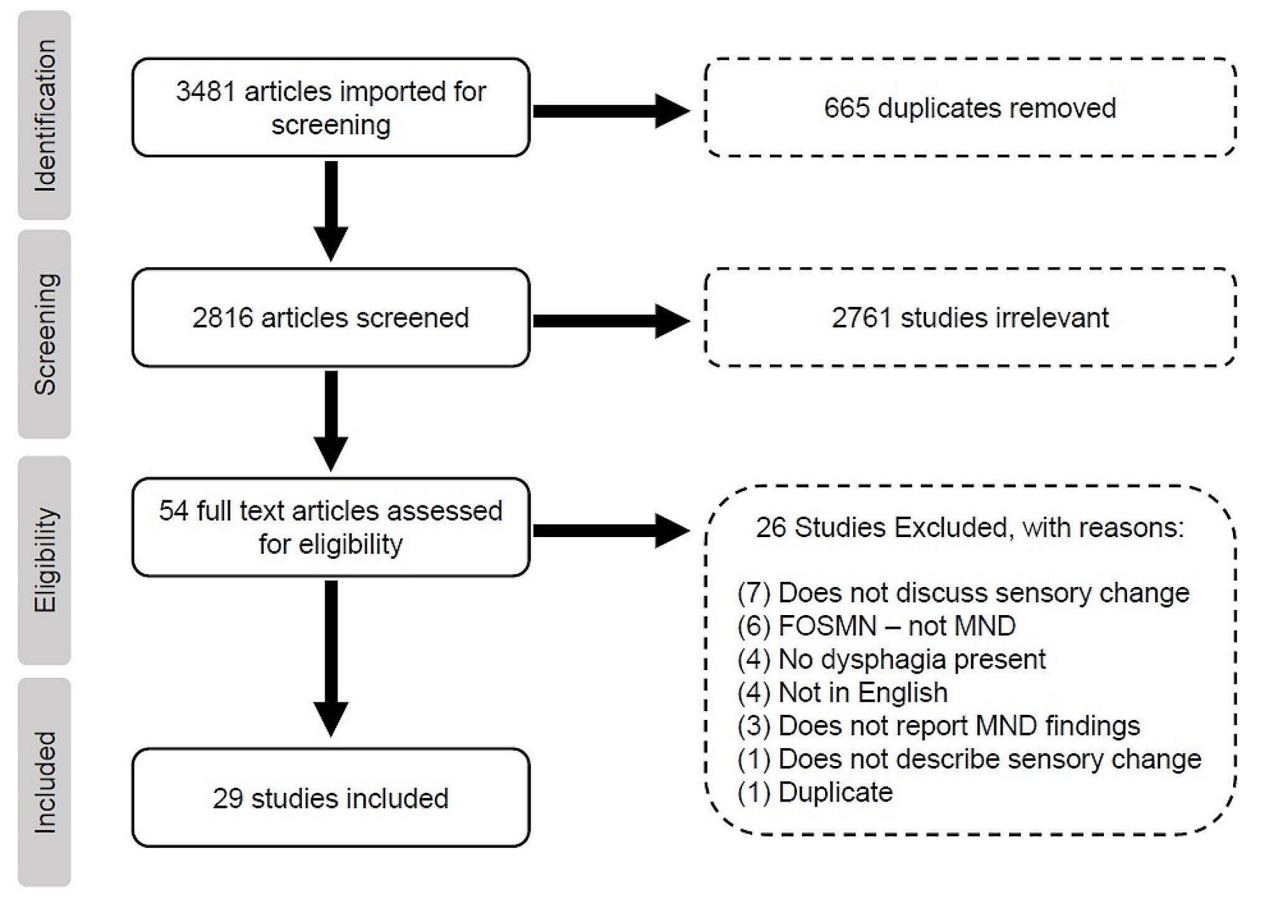
Preferred reporting items for systematic reviews and meta-analyses (PRISMA) flowchart. *FOSMN* facial onset sensory and motor neuronopathy

A total of 3,481 studies were initially imported into the Covidence platform [33]. After deduplication, 2,816 articles underwent title and abstract screening. Of these, 2,761 were excluded as they did not meet inclusion criteria, leaving 54 studies that progressed to full text review. Of these, 29 studies met inclusion criteria (Fig. 1). A summary of these studies is provided in Table 2.

### Study Design and Evidence Grading

Study designs varied across the 29 included studies, including observational (*n* = 16), case reports (*n* = 4), expert opinion (*n* = 4), commentary (*n* = 2), literature reviews (*n* = 2), and a scoping review (*n* = 1). Details of these designs and their GRADE ratings are summarised in Table 2.

**Table 2:** Study designs and GRADE ratings

Study reference	Study design	GRADE Rating
Amin et al. [4]	Cross-sectional	Low
Ashray, Patel, & Hirsch [39]	Case study	Very low
Bin Ayaz et al. [40]	Case study	Very low
Carvajal-González & Iglesias [41]	Case study	Very low
Gibbons et al. [38]	Cross-sectional	Moderate
Graner & Strand [55]	Expert opinion	Very low
Gunther et al. [44]	Case-control	Low
Hadjikoutis et al. [37]	Literature review	Moderate
Hairong [56]	Commentary	Very low
Hughes & Wiles [36]	Case-control	Low
Ikeda [42]	Commentary	Very low
Jesus et al., [52]	Cross-sectional	Low
Lang, Schwandner, & Hecht [49]	Case-control	Low
Omari et al. [53]	Case-control	Moderate
Onesti et al. [43]	Retrospective cross sectional	Low
Orrell [57]	Expert opinion	Very low
Pelletier et al. [5]	Case-control	Low
Petzold, Einhäupl, & Valdueza [50]	Case series	Very low
Piretta et al. [51]	Cross-sectional	Low
Ramroop & Cruz [58]	Expert opinion	Very low
Robison et al. [54]	Cross-sectional	Low
Ruopollo et al. [6]	Cross-sectional	Low
Ruopollo et al. [10]	Cross-sectional	Low
Saramago & Franceschi [45]	Literature review	Low
Tabor-Gray et al. [7]	Case-control	Moderate
Tarlarini et al. [46]	Cross-sectional	Low
Viguera et al. [47]	Case-control	Low
Waito et al. [8]	Scoping review	High
West [48]	Expert opinion	Very low

These data show an overall low level of methodological rigour within the included literature. Articles reporting no changes in sensory function most commonly had a very low GRADE rating (very low 66.67%, low 22.22%, moderate 11.11%), while those describing the presence of sensory changes were most frequently rated as low (very low 20%, low 60%, moderate 15%, high 5%).

### Summary of Sensory Findings

**Fig. 2:**
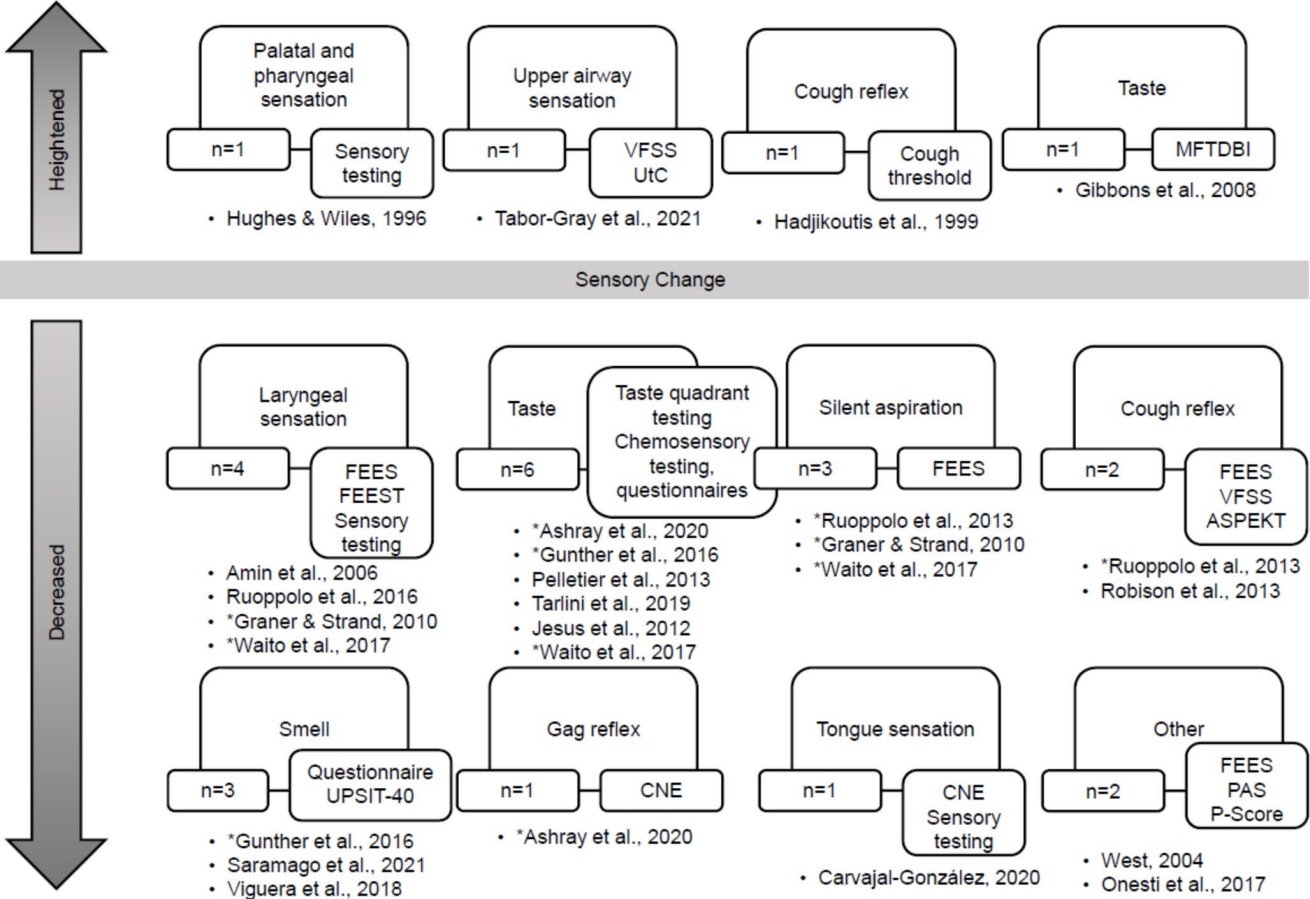
Included studies characterised by their heightened or decreased sensation type and outcome measures used. *Note.* * Represents studies that report findings for multiple sensation types. *VFSS* Videofluoroscopic Swallow Study, *UtC* Urge to Cough, *MFTDBI* the Manchester Frontotemporal Dementia Behavioural Interview, *FEES* Fibreoptic Endoscopic Evaluation of Swallowing, *FEEST* Flexible Evaluation of Swallowing with Sensory Testing, *ASPEKT* Analysis of Swallowing Physiology: Events, Kinematics, and Timing, *CNE* Cranial Nerve Examination, *PAS* Penetration Aspiration Scale, *P−Score* Pooling Score, *UPSIT−40* University of Pennsylvania Smell Identification Test

Of the included 29 studies, 20 reported the presence of sensory changes whereas nine reported no changes in sensory function. Sensory changes relating to swallowing included laryngeal tactile sensation (*n* = 4), taste (*n* = 7), gag reflex (*n* = 1), cough reflex (*n* = 4), tongue sensation (*n* = 1), smell (*n* = 3), palatal and pharyngeal tactile sensation (*n* = 1), silent aspiration (*n* = 3), and undefined sensory changes of the swallowing mechanism (*n* = 2). Most frequently, sensory changes were described as ‘decreased’, or presenting as diminished when compared to expected ranges (*n* = 20) (Fig. 2). Notably, four studies reported heightened sensory function in palatal and pharyngeal tactile sensation [36], upper airway sensation to tussigenic irritants [7], the cough reflex [37], and taste [38] (Fig, 2). Of these studies, three reported on primary findings from original experiments, while one included secondary data from others’ research with citation. This indicates a similar level of methodological rigour to the 16 studies finding reduced sensation with 12 including primary findings and four referencing secondary data with citations to the original work. Of the studies reporting no sensory change, four included primary findings and five included secondary data. Of these secondary data, four statements included reference to the original work, indicating a lower level of methodological rigor and, consequently, lower validity and reliability.

### Demographic Information

Sample sizes ranged from one [39–42] to 145 plwMND [43]. Overall, a total of 455 male, 336 female, and 51 unspecified gendered MND participants were reported on in the included literature, with a mean age across all studies of 62.42 (SD 5.7) years. All participants were reported to have a diagnosis of MND; however, the diagnostic process was frequently not described.

Included studies were undertaken across a diverse range of countries, with the highest frequency from the United States of America (*n* = 9), followed by Italy (*n* = 5), Germany (*n* = 3), and the United Kingdom (*n* = 3). Year of publication ranged from 1996 to 2022, with 90% of the studies (*n* = 18) having been published since 2013.

### Study Aims

Study aims were diverse, with 15 of 29 studies exploring sensation related to swallowing in plwMND specifically. Of these, twelve studies observed sensory changes [4–7, 36, 37, 39, 44–48], while three did not [49–51]. The remaining fourteen studies did not evaluate sensation in a swallowing-specific context and had varying aims. Nine studies did not explicitly aim to explore sensation, however, included findings from sensory assessments [10, 38, 40–43, 52–54]. The remaining five studies did not actively investigate sensory changes, however, provided a commentary on its presence or absence [8, 55–58]. Of the fourteen studies that did not evaluate sensation in the context of swallowing, eight reported observing sensory changes [8, 10, 38, 41, 43, 52, 54, 55], while six did not [40, 42, 53, 56–58].

### Outcome Measures

All studies reported on unique outcome measures, such that it was impossible to identify patterns across more than one study. Studies included objective (*n* = 3), subjective (*n* = 5), mixed (*n* = 12), or no outcome measures (i.e., where only a commentary was provided) (*n* = 8). The most frequently used outcome measures were questionnaires (*n* = 6), Fibreoptic Endoscopic Evaluation of Swallowing (FEES) (*n* = 5), and the Amyotrophic Lateral Sclerosis Functional Rating Scale Revised (ALSFRS-R) (*n* = 5). These outcome measures are detailed in Table 3.

**Table 3:** Summary of findings reported by included studies

Study details				Sensory outcomes		Outcome measures	
Author (year)	n=	Location	Aim	Sensation type	Change reported	Objective	Subjective
**Sensory change reported (** ***n = 20)***							
Amin [4]	22	USA	Sensory	Laryngeal sensation	Decrease	• FEEST • FEES • Reflux measures	• Dysphagia questionnaire
Ashray [39]	1	Cyrpus	Sensory	(a) Whole mouth sensation (b) Gag reflex	(a) Decrease (b) Decrease	• Neurological exam • MRI • EMG • CNE • Fungiform papillae count	• Chemosensory testing • Taste quadrant testing
Carvajal-González [41]	1	Columbia	Non-sensory	Tongue sensation	Decrease	• CNE • Motor and sensory exam • MRI	Nil
Gibbons [38]	16	UK	Non-sensory	Taste	Increase	• SPECT	• The Manchester FTD Behavioural Interview
Graner [55]	Nil	USA	Non-sensory	(a) Laryngeal sensation (b) Silent aspiration	(a) Decrease	Nil	Nil
Gunther [44]	90	Germany	Sensory	(a) Taste (b) Smell	(a) Decrease (b) Decrease	Nil	• NMSQuest
Hadjikoutis [37]	Nil	UK	Sensory	Cough reflex	Increase	Nil	Nil
Hughes [36]	171	UK	Sensory	Palatal and pharyngeal sensation	Increase	• Neurological exam	• Swallowing questionnaire • Sensory testing of taction to the palate and posterior pharyngeal wall
Jesus [52]	40	France	Non-sensory	Taste	Decrease	• The Salle Test • The DiPippo Test	• Nutrition assessment • Taste perception and dysphagia questionnaire
Onesti [43]	145	Italy	Non-sensory	Swallowing mechanism sensation	Decrease	• FEES • PAS • P-SCA	• ALSFRS-R
Pelletier [5]	16	USA	Sensory	(a) Taste (intensities) (b) Taste (perception)	(a) Decrease (b) No Change	Nil	• The Spatial Taste Test
Robison [54]	100	USA	Non-sensory	(a) Cough reflex (b) Silent aspiration	(a) Decrease	• VFSS • PAS • ASPEKT	• ALSFRS-R
Ruoppolo [6]	114	Italy	Sensory	(a) Laryngeal sensitivity (b) Silent aspiration	(a) Decrease	• FEES • Laryngeal sensitivity testing • PAS • P-Score • MRC scale for grading muscle strength • Epiglottis biopsy	• ALSFRS-R
Ruoppolo [10]	49	Italy	Non-sensory	Cough reflex	Decrease	• FEES • Anterior tongue obility and oral sensitivity testing	• Patient’s self-reported experience of dysphagia
Saramago [45]	Nil	USA	Sensory	Smell	Decrease	Nil	Nil
Tabor-Gray [7]	32	USA	Sensory	(a) Cough reflex (b) Silent aspiration	(a) Decrease	• VFSS • PAS	• UtC • Cough reflex testing
Tarlarini [46]	32	Italy	Sensory	Taste	Decrease	Nil	• ALSFRS-R • Taste perception questionnaires
Viguera [47]	78	USA	Sensory	Smell	Decrease	• UPSIT-40 • FVC • ALS-CBS	• ALSFRS-R • Background history questionnaire
Waito [8]	Nil	Canada	Non-sensory	(a) Laryngeal sensation (b) Taste (c) Silent aspiration	(a) Decrease (b) Decrease	Nil	Nil
West [48]	Nil	USA	Sensory	Swallowing mechanism sensation	Decrease	Nil	Nil
**No Sensory Change Reported (** ***n = 9)***							
Bin Ayaz [40]	1	Pakistan	Non-sensory	General sensation	No change	Nerve conduction study	Nil
Hairong [56]	2	China	Non-sensory	General sensation	No change	Nil	Nil
Ikeda [42]	1	Japan	Non-sensory	General sensation	No change	Unspecified	Unspecified
Lang [49]	26	Germany	Sensory	(a) Taste (b) Smell	(a) No change (b) No change	Nil	• Sniffin’ Sticks • Taste Strips
Omari [53]	11	Australia	Non-sensory	Taste	No change	• VFSS • Manometry	Nil
Orrell [57]	Nil	UK	Non-sensory	General sensation	No change	Nil	Nil
Petzold [50]	2	Germany	Sensory	Taste	No change	Nil	• Spatial gustatory function testing
Piretta [51]	20	Italy	Sensory	Taste	No change	• FEES • Phoniatric examination	• SDQ
Ramroop [58]	Nil	USA	Non-sensory	General sensation	No change	Nil	Nil

*FEEST* Flexible Endoscopic Evaluation of Swallowing with Sensory Testing, *FEES* Fibreoptic Endoscopic Evaluation of Swallowing, *MRI* Magnetic Resonance Imaging, *EMG* Electromyography, *SPECT* single−photon emission computerized tomography, *FTD* Frontotemporal Dementia, *NMSQuest* Non−Motor Symptoms Questionnaire, *VFSS* Videofluoroscopic Swallow Study, *ALSFRS−R* Amyotrophic Lateral Sclerosis Functional Rating Scale Revised, *PAS* Penetration Aspiration Scale, *P−SCA* Pooling with Sensation, Collaboration, and Age, *SDQ* Swallowing Dysfunction Questionnaire, *ASPEKT* Analysis of Swallowing Physiology: Events, Kinematics, and Timing, *P−Score* Pooling Score, *MRC* Medical Research Council, *UtC* Urge to Cough, *UPSIT−40* University of Pennsylvania Smell Identification Test, *FVC* forced vital capacity, *ALS−CBS* Amyotrophic Lateral Sclerosis Cognitive Behavioural Screen, *CNE* cranial nerve exam

## Discussion

This scoping review mapped and summarised the current literature describing sensory changes related to swallowing in MND, with a growing body of evidence reporting the presence of sensory changes in this population. Swallowing-related sensory function was generally described as decreased, with only a few studies (*n* = 4) reporting heightened sensation related to palatal and pharyngeal sensation, upper airway sensation, the cough reflex, and taste [7, 36–38]. Across the included studies, outcome measures assessed, and results reported were heterogeneous and characterised by poor reporting of clinically significant data.

### Taste and Smell

Taste dysfunction in MND was described as ageusia of the tongue and palate, lower taste intensities, or loss in taste perception [5, 8, 44, 46, 52]. In addition, changes to olfactory perception were also reported [44, 45, 47]. Reduction of both taste and smell in plwMND may affect swallowing safety and efficiency as both have been shown to influence typical swallowing physiology. Indeed, strong flavours and olfactory stimuli are used clinically in the management of dysphagia [51, 59–62]. As such, taste and smell changes may need to be considered in the clinical assessment of swallowing in plwMND.

Beyond impacting swallowing physiology, the integration of gustatory and olfactory inputs also plays an important role in the control of appetite [63]. As such, altered processing of taste and smell stimuli may lead to reduced nutritional intake and consequently, weight loss [64]. This is important to consider as Body Mass Index (BMI) has been shown to be a predictor of life expectancy in MND [65].

An additional impact on appetite may include changes to saliva. plwMND may experience difficulty managing secretions resulting in excess saliva (sialorrhea) [66]. For example, 50% of plwMND have been estimated to experience impaired saliva production [67], with 17% reporting moderate-severe excess saliva [68], and 10% experiencing severe sialorrhea [69]. In addition, 9.1% of plwMND reported xerostomia, or dry mouth, which can result in a tenacious saliva [70]. As salivary changes may modify taste perception and hence influence appetite and eating-related pleasure, saliva status, taste and appetite should all be considered during swallowing-related assessment in plwMND.

### Cough Reflex and Gag Reflex

Sensory function is not only critical for the initiation, execution and modulation of swallowing-related biomechanics, but also contributes to airway protection mechanisms. As part of these mechanisms, the cough reflex plays a vital role in expelling foreign material from the airway, consequently limiting aspiration and reducing risk for aspiration pneumonia [37]. Similarly, the gag reflex can prevent substances from prematurely entering the pharynx, larynx, or trachea [71]. Growing evidence has highlighted the reduction or absence of gag and cough reflexes in MND [7, 10, 39, 54]. In the context of reports that document the presence of silent aspiration [8, 10, 55] in plwMND, cough reflex testing should be considered in swallowing examination in MND. Future studies may also benefit from investigation into the differences in cough reflex across MND phenotypes related to the presence or absence of upper motor neuron involvement and hyperreflexia. At present, this analysis is unable to be completed due to the unspecific and heterogeneous language used to describe MND phenotypes in the included literature.

Impaired tactile sensation in the larynx [4, 6, 8, 42], pharynx [36], and upper airway [54] may also contribute to the prevalence of impaired cough reflex [10, 37, 54] and gag reflex [39] in this population. Despite the presence or absence of a gag reflex not being an indicator of dysphagia [71], this information further lends to the necessity for sensory investigation of structures involved in swallowing during swallowing examinations in MND, with continued monitoring throughout the disease process to ensure swallow safety.

### Somatosensory Function

Bolus properties have been shown to impact a variety of swallowing metrics, including upper oesophageal sphincter (UES) opening duration and opening extent, pharyngeal peak pressure, and hypopharyngeal intrabolus pressure [72]. The included literature describes a range of changes to tactile sensation in plwMND including reduced sensitivity to tactile stimuli in the larynx [4, 6, 8, 42], palate and pharynx [36], and tongue [41]. Reduced sensitivity in these areas may impact detection of somatosensory information relating to the properties of a bolus, including its size, consistency, temperature, weight, and velocity, which heavily influences swallowing physiology across all phases of swallowing [73, 74]. These bolus properties have been shown to impact a variety of swallowing biomechanical metrics, including UES opening duration and opening extent, pharyngeal peak pressure, and hypopharyngeal intrabolus pressure [72].

Further, the use of analgesics for pain management in plwMND may pose an additional cause for changes to somatosensory function and subsequent changes to swallowing biomechanics. Analgesics that inhibit the somatosensory system, including changes to perception of temperature, are suggested for use in plwMND to manage muscle pain [75–77], and have been linked to increased aspiration risk [78]. Thus, review of current medications and their potential impact to swallow biomechanics should be considered when assessing and managing dysphagic plwMND.

### Implications for Swallow Safety

This review revealed a growing body of literature reporting changes to smell, taste, the cough and gag reflexes, and somatosensory function in plwMND, all of which are relevant for safe and efficient swallowing. Beyond their impacts on swallowing biomechanics, changes to these sensory functions are likely to contribute, at least in part, to reports of absent laryngeal adductor reflex [6], or the high incidence of silent aspiration in this population [8, 10, 55].

It is, therefore, important to consider how sensory changes may interact with swallowing function, especially in the context of likely impairment of swallowing-related motor function in MND. For example, in non-impaired motor systems, increased motor output may compensate for changes in sensory function [79–90]. Reduced motor function in MND may limit such compensatory mechanisms for sensory changes in addition to the decline in swallowing motor function driven by degeneration of the motor system. As such, swallowing safety and efficiency in plwMND may be at risk from both sensory *and* motor changes, which need to be considered in swallowing assessment and management.

*Interaction of sensory changes reported in MND with natural ageing process.*

It is important to recognise that the changes in sensory function in MND collated in this review may, at least in part, also be related to the natural ageing process. With the average age of onset of MND being 58 years [91], natural sensory decline as the result of advancing age poses a potential alternative cause for the sensory loss described in plwMND. The natural aging process is associated with a range of impacts on sensations relating to swallowing, including taste [79, 81–83, 88, 89, 92], smell [80, 85, 87, 90], and the cough reflex [84, 86], all of which have been implicated in plwMND. It should be noted, however, that while sensory changes have been reported as part of the natural ageing process, these changes are more likely to occur at an older age than the average age of onset of MND (58 years). In addition, plwMND have been reported to experience sensory changes that are not typically described in the literature as occurring within the natural ageing process, including changes to tactile sensation of the palate, pharynx, larynx, upper airway, and tongue. Therefore, on balance, we propose that the changes to sensory function summarized here are more likely to be related to MND-specific disease processes than ageing.

### A Comment on Changes in Sensory Function in Frontotemporal Dementia (FTD) and Facial Onset Sensory and Motor Neuronopathy (FOSMN)

A pertinent theme established in the included literature was that of MND being a “multisystem disorder”, implicating systems other than only the motor system. One such example of this is found in the growing links between MND and frontotemporal dementia (FTD) [93, 94]. It is estimated that 15% of plwMND will develop a frank FTD [94]. People living with FTD have been observed to experience impaired olfaction [95–97], similar to that described in the included literature pertaining to plwMND. This highlights a key clinical consideration for sensory testing in dysphagic plwMND, particularly when FTD is present or suspected.

An additional consideration relates to the emerging literature discussing a disease that features sensory disturbance with potential links to MND – facial onset sensory and motor neuronopathy (FOSMN) [98–100]. While the etiological and pathophysiological mechanisms underpinning FOSMN remain unclear, it poses a potential phenotype of MND of which sensory dysfunction appears to be a hallmark feature. Similar to MND, dysphagia is frequently reported in people living with FOSMN [99, 101–103]. Taste disturbance, absent gag reflex, and absent palatal and pharyngeal reflexes have also been reported in this population [98, 99, 101, 102].

Exploration of the roles of FTD and emerging MND phenotypes in the presence of sensory disturbance in plwMND was not the focus of this study. However, specific evaluations of these conditions require further research.

### Limitations

This review included only articles in English. We acknowledge that this may result in relevant literature that was published in a different language being excluded. The included literature in this scoping review is characterized by heterogeneity in its outcome measures and methods, impacting our ability to establish patterns of sensory change in plwMND. Furthermore, many studies included data that was not objectively assessed, with no baseline measurements available, resulting in low research reliability. Demographic and clinically significant information, such as disease severity and onset, dysphagia severity and onset, and consistency of food and liquid trials, were frequently not discussed in the included literature. This worked to further compound the difficulty in drawing clinical conclusions from the literature.

Additionally, very few studies took multiple measurements or replicated their results. As such, data reported in the included literature are likely to have low validity due to unknown levels of variability as well as low reliability. It is acknowledged that the reason for single measurements likely relates to (i) limited resources, (ii) accelerated disease degeneration impacting ability to complete multiple assessments, and (iii) limited life expectancy.

Due to the heterogeneity in study designs and outcome measures reported, we were unable to describe universally applicable patterns of sensory change in MND. However, here we suggest key areas to consider within both clinical practice and future research.

### Clinical Practice and Research Implications

While the current clinical guidelines for MND management make reference to the importance of swallowing assessment and management, they do not discuss how sensation should be considered in this process [76, 104]. Based on the findings of this review, we propose that the assessment and management of swallowing function in plwMND should, as individually appropriate, consider changes to olfactory, gustatory, and somatosensory function related to swallowing. Further, the impact of these changes on swallowing safety mechanisms, along with psychosocial implications, should continue to be monitored throughout disease progression.

Overall, this review highlights limited and contradictory knowledge regarding sensory changes related to swallowing in MND. The generally low quality of evidence in this area is a call to arms for thorough, high-quality investigations into sensory changes in MND. Research using control groups, longitudinal testing, and consistent evidence-based outcome measures is required to confirm the presence of sensory deficits related to swallowing in plwMND. Replicating existing studies with the same outcome measures may improve validity and reliability of the data. In addition, the included literature subjectively describes the degree of sensory change present, often in comparison to healthy cohorts. Studies have not yet, however, taken steps to accurately describe the effect size. This limits our ability to estimate the presence and magnitude of sensory changes in plwMND. Finally, we suggest that investigations into how sensory deficits are contributing to, and interact with, dysphagia in plwMND are also warranted.

## Conclusion

The high prevalence of dysphagia in plwMND poses serious health and psychosocial implications for this population [2, 8–10, 13, 16, 18]. Given the established significance of sensation in swallowing and swallowing safety [6, 37, 51, 59–62], an understanding of how sensory function may be impacted by MND is necessary to ensuring appropriate management of dysphagia in plwMND. The review presented here identified a growing body of evidence reporting sensory changes related to safe and efficient swallowing in plwMND. Of note, there is a paucity of literature, which is contradictory and characterised by low methodological rigour. This highlights the need for further high-quality research. Until more conclusive evidence is established related to sensory changes in MND, the data synthesised in this review suggest that sensory changes should be considered in the clinical assessment and management of dysphagic plwMND.
